# Subclinical pulmonary abnormalities in juvenile dermatomyositis

**DOI:** 10.1186/1546-0096-12-S1-P97

**Published:** 2014-09-17

**Authors:** Adriana M Sallum, Douglas Coutinho, Claudine Veiga, Lisa Suzuki, Luiz Antonio Nunes, Clovis A Silva, Joaquim Rodrigues

**Affiliations:** 1 Pediatric Rheumatology Unit - Pediatrics Department , Instituto da Criança - HC FMUSP, São Paulo, Brazil; 2 Pediatric Pulmology Unit -Pediatrics Department, Instituto da Criança - HC FMUSP, São Paulo, Brazil; 3 Pediatric Radiology Unit - Pediatrics Department , Instituto da Criança - HC FMUSP, São Paulo, Brazil

## Introduction

Pulmonary involvement in juvenile dermatomyositis (JDM) is frequent and associated with poor outcome. However, a systematic assessment of pulmonary function and high-resolution computed tomography (HRCT) was rarely reported in this population.

## Objectives

To assess pulmonary function and HRCT in JDM patients and to evaluate possible associations between pulmonary abnormalities and disease activity, cumulative damage and health-related quality of life (HRQL) scores.

## Methods

A cross-sectional study was performed in 20 JDM patients. Pulmonary function test included spirometry, body plethysmography and diffusion capacity for carbon monoxide (DLCO). They were also carried out six-minute walk test (6MWT) and HRCT scan. Disease activity score (DAS), childhood myositis assessment scale (CMAS), myositis damage index (MDI) and HRQL (Pediatric Quality of Life Inventory - PedsQL) data were also assessed

## Results

The mean age was 11.6 years (6-18). Subclinical mild/moderate obstruction according to American Thoracic Society criteria was observed in 35% and DLCO was reduced in 20% of JDM patients. Spirometric and/or DLCO abnormalities were observed in 45% of patients. In plethysmography, reduced total lung capacity (TLC) and conductance were observed in 25% and 50% of JDM patients, respectively. In contrast, increased resistance and residual volume (RV)/TLC were evidenced in 10% and 35% of JDM patients, respectively. Thirteen patients underwent HRCT and 8 had alterations: interstitial lung disease in 6 and a mixed pattern in two. A positive correlation was observed between DAS and ratio between forced expiratory volume in one second and vital capacity (VEF1/CV) (r=+0.50, p=0.003), conductance (r=+0.46, p=0.045) and HRCT score (r=+0.60, p=0.003). A positive correlation was observed between CMAS and VEF1/CV (r=+0.47, p=0.042), DLCO (r=+0.67, p=0,002) and 6MWT (r=+0.54, p=0.048), and negative correlation between DAS and HRCT score (r=-0.63, p=0.021). Correlations were identified between MDI and conductance (r=+0.72, p=0.0004), DLCO (r=-0.46, p=0.042) and HRCT score (r=+0,81, p=0.0008); and between PedsQL and VEF1/CV (r=+0.45, p=0.046), conductance (r=-0.60, p=0.006) and HRCT score (r=+0.62, p=0.024). Correlations were also observed between HRCT score and VEF1/CV (r=-0.64, p=0.017), forced expiratory flow between 25 and 75% of vital capacity (FEF25%>75 %) (r=-0.59, p=0.035) and conductance (r=+0.78, p=0.0018).

## Conclusion

Subclinical pulmonary abnormalities were frequent in this rare idiopathic inflammatory myopathy. Importantly, these findings may be related to disease severity and activity, and may influence HRQL of these patients.

## Disclosure of interest

None declared.

